# National survey of China's oncologists' knowledge, attitudes, and clinical practice patterns on complementary and alternative medicine

**DOI:** 10.18632/oncotarget.14560

**Published:** 2017-01-09

**Authors:** Geliang Yang, Richard Lee, Huiqing Zhang, Wei Gu, Peiying Yang, Changquan Ling

**Affiliations:** ^1^ Department of Integrative Oncology, Changhai Hospital, Second Military Medical University, Shanghai, China; ^2^ Department of Medicine, University Hospitals Case Medical Center and Case Western Reserve University, Cleveland, Ohio, USA; ^3^ Department of Palliative, Rehabilitation and Integrative Medicine, The University of Texas MD Anderson Cancer Center, Houston, Texas, USA

**Keywords:** oncologists, complementary and alternative medicine, national survey, knowledge, attitudes

## Abstract

It is common for cancer patients to use complementary and alternative medicine (CAM). This study was designed to explore China's oncologists’ knowledge, attitudes and clinical practices regarding CAM use by their patients. An online survey was conducted of China's oncologists. Among 11,270 participants who completed the online survey, 6,007 (53.3%) were identified as oncologists. Most were men (75.2%), with a mean age of 33.4 (standard deviation: 6.5) years. The 6,007 oncologists discussed with 36.5% of their patients about CAM. Most of them (75.6%) did not want to initiate discussions due to lack of knowledge on CAM. Oncologists estimated that 40.0% of their patients used CAM treatments. Oncologists reported that 28.7% of their patients underwent anticancer therapy with the concurrent use of CAM. Four out of five of the responding oncologists self-reported inadequate knowledge and only 22.0% reported receiving professional education on CAM. Nearly half (44.9%) of the oncologists believed CAM treatment was effective for symptoms and treatment of cancer. Physician factors associated with initiating discussions with patients about CAM use included sex, age (≥ 33 years), medical license for traditional Chinese medicine, enough knowledge and professional education experience. China's oncologists infrequently discussed with their patients about CAM due to lack of knowledge. Most of the oncologists did not encourage CAM use.

## INTRODUCTION

A national survey of cancer prevalence by the National Central Cancer Registry of China indicated that an estimate of 4.3 million new cancer cases and about 2.8 million cancer deaths would occur in China in 2015 [1]. Interest in the use of complementary and alternative medicine (CAM) has grown rapidly in the past decade, in view of dissatisfaction with conventional anticancer treatments, Internet marketing, patients’ desire to maintain a high quality of life and control over their health care decisions [2–3].

In 2007, almost 40% of adults had used CAM therapy in the United States [4]. The popularity of CAM use among cancer patients has been reported in different countries, however, studies have documented limited communication and discrepant views between cancer patients and oncologists regarding CAM [5–9]. A national survey study of US oncologists published by Lee et al. showed that less than one half of oncologists initiated discussions with patients about herb and supplement use and nearly two thirds of oncologists self-reported a lack of knowledge and education about herbs and supplements [10]. These types of products have been found to have treatment-related toxicity and may interact with medications including chemotherapies [11–14].

Of the different CAM therapies, traditional Chinese medicine (TCM) is a well-recognized CAM modality. In China, TCM has evolved over thousands of years with a standardized system of theories, diagnostics and therapies, and has been widely used for anticancer treatment for a long time [15–16]. However, whether and how oncologists discuss the use of CAM with their patients remain unclear in China. The purpose of this study was to explore China's oncologists’ knowledge, attitudes and clinical practice regarding CAM using a national online cross-sectional survey in 2015.

## RESULTS

**Figure 1:**
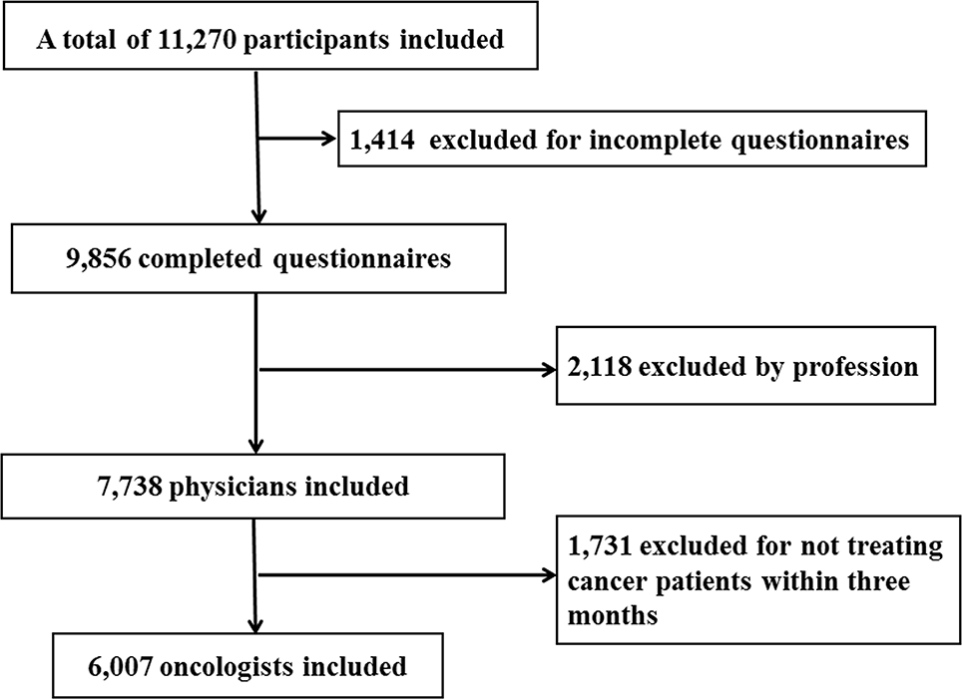
Flowchart for inclusion and exclusion of study population

Totally 11,270 participants responded the online survey, 1,414 (12.5%) failed to complete the survey. Among the 9,856 completed questionnaires, 2,118 were not answered by physicians (they were answered by medical students, or pharmacists, or nurses, or others). Then in the remaining 7,738 physicians, 1,731 did not treat any cancer patients in the past three months. A total of 6,007 physicians were identified as oncologists which represents over half (53.3%) of respondents (Figure 1).

The average age of oncologists was 33.4 years old (standard deviation (SD), 6.5). The majority of the oncologists (75.2%) were male. See Table 1. The distributions of their regions were 16.4% in metropolitan areas, 38.7% in provincial capitals and 44.8% in other cities. The oncologists predominantly practiced in a general hospital (52.4%) and an academic hospital (40.4%) with a mean working year of 6.1 years (SD, 6.1). Medical oncologist, surgical oncologist and radiologist oncologists comprised 41.9%, 32.2% and 5.1% of the respondents, respectively. Most of the oncologists (84.1%) indicated that they had a medical license for clinical medicine while 15.9% had a medical license for TCM. Three-fourths of oncologists reported personal use of CAM therapies.

**Table 1:** Characteristics of survey respondents (*n* = 6,007)

Characteristics	Mean ± SD (year)	No. (%)
Age (year)	33.4 ± 6.5	
< 30		1,999 (33.3)
30–39		2,948 (49.0)
40–49		920 (15.3)
> 50		140 (2.3)
Sex		
Male		4,517 (75.2)
Female		1,490 (24.8)
Region		
Metropolitan areas*		987 (16.4)
Provincial capitals		2,326 (38.7)
Other cities		2,694 (44.8)
Practice setting		
General hospital		3,149 (52.4)
Academic hospital		2,424 (40.4)
Community hospital		168 (2.8)
Private hospital		163 (2.7)
Other		103 (1.7)
Specialty		
Medical oncologist		2,516 (41.9)
Surgical oncologist		1,936 (32.2)
Radiologist		308 (5.1)
Psychotherapist/psychiatrist		270 (4.5)
Hematologist		104 (1.7)
Pediatric oncologist		77 (1.3)
Stomatologist		48 (0.8)
Other		748 (12.5)
Working duration (year)	6.1 ± 6.1	
< 10		4,571 (76.1)
≥ 10		1,436 (23.9)
Type of medical license		
Clinical medicine		5,053 (84.1)
Traditional Chinese medicine		954 (15.9)
Personal use of CAM		
Yes		4,572 (76.1)
No		1,435 (23.9)

SD: standard deviation; CAM: complementary and alternative medicine.

*Sample included one oncologist in Hongkong and two oncologists in Macau.

Oncologists self-reported discussing with 36.5% (SD = 26.8 and median = 30) of their patients about CAM. Approximate two-thirds (67.2%) of the oncologists indicated a desire to initiate a discussion about CAM with the patients. The remaining oncologists (32.8%) who did not want to initiate the discussions, the majority of them (75.6%) attributed the reason to the fact that they knew little about CAM, followed by limited-time for discussion (9.5%), not believing in CAM (5.7%) and no interest in use of CAM (5.2%). When asked about CAM by the patients, 55.8% of the oncologists would remain neutral about the use of CAM. When presented with the patients’ disclosure that they were using or would use CAM, 62.8% of the oncologists would keep neutral about CAM use and only 35.9% would encourage CAM use. See Table 2.

**Table 2:** Oncologists’ communication patterns with patients regarding CAM (*n* = 6,007)

Pattern	Mean ± SD, Median (%)	No. (%)
Please estimate the percentage of your patients that have discussed with you the topic of CAM?	36.5 ± 26.8, 30	
Do you want to initiate the discussions with your patients about CAM?		
Yes		4,036 (67.2)
No		1,971 (32.8)
What is the reason for not initiating the discussions with your patients about CAM? (*n* = 1,971)*		
Know little about CAM		1,490 (75.6)
imited-time consultation		188 (9.5)
Do not believe in CAM		113 (5.7)
No interest in use of CAM		103 (5.2)
Other		77 (3.9)
How do you respond if asked about CAM by your patients?		
Neither encourage nor discourage		3,354 (55.8)
Encourage to continue		2,287 (38.1)
Advise to stop		174 (2.9)
Other		192 (3.2)
How do you react to your patients who disclose that they are using or will use CAM?		
Neither encourage nor discourage		3,773 (62.8)
Encourage to continue		2,158 (35.9)
Advise to stop		76 (1.3)

CAM: complementary and alternative medicine; SD: standard deviation.

*Sample included oncologists who do not want to initiate the discussions with patients about CAM (*n* = 1,971).

On average, the oncologists estimated that 40.0% (SD = 28.7) of their patients used CAM treatments versus 60.0% (SD = 30) among the 954 oncologists who possessed TCM medical license. Most (83.7%) of the oncologists cited improving immune system as the main reason for CAM use by patients. They reported that 28.7% (SD = 26.6 and median = 20) of their patients underwent anticancer therapy with concurrent use of CAM. Chinese herbal medicine (66.2%) was the most commonly used CAM therapy, and the oncologists may give priority to CAM use when their patients were suffering from the most common symptoms such as lack of appetite (68.6%), fatigue (62.8%) and sleep disorder (60.1%) (Table 3).

**Table 3:** Oncologists’ clinical practice patterns with patients regarding CAM (*n* = 6,007)

Pattern	Mean ± SD, Median (%)	No. (%)
Please estimate the percentage of your patients that have used or currently use CAM?	40.0 ± 28.7, 30	
The reason for CAM use that you recommend to your patients?		
Improve immune system		5,030 (83.7)
Improve quality of life		3,976 (66.2)
Manage symptoms		3,253 (54.2)
Increase the effect of conventional treatment		2,769 (46.1)
Cure disease		1,436 (23.9)
Other		273 (4.5)
No recommendation in use of CAM		185 (3.1)
Please estimate the percentage of your patients that received chemotherapy/targeted therapy with the concurrent use of CAM?	28.7 ± 26.6, 20	
What type of CAM you may recommend to your patients for treatment?		
Chinese herbal medicine		3,975 (66.2)
Dietary therapy		2,495 (41.5)
Acupuncture		1,305 (21.7)
Tai chi		947 (15.8)
Qi gong		534 (8.9)
Massage therapy		510 (8.5)
Other		235 (3.9)
No recommendation in form of CAM		392 (6.5)
What symptom your patients are suffering from, you may give priority to CAM use?		
Lack of appetite		4,119 (68.6)
Fatigue		3,770 (62.8)
Sleep disorder		3,608 (60.1)
Excess sweating		3,328 (55.4)
Abdominal distension		2,213 (36.8)
Nausea/vomiting		1,882 (31.3)
Dry mouth		1,724 (28.7)
Numbness/tingling		1,199 (20.0)
Pain		1,128 (18.8)
Other		343 (5.7)
No recommendation in use of CAM		337 (5.6)

CAM, complementary and alternative medicine; SD, standard deviation.

**Figure 2:**
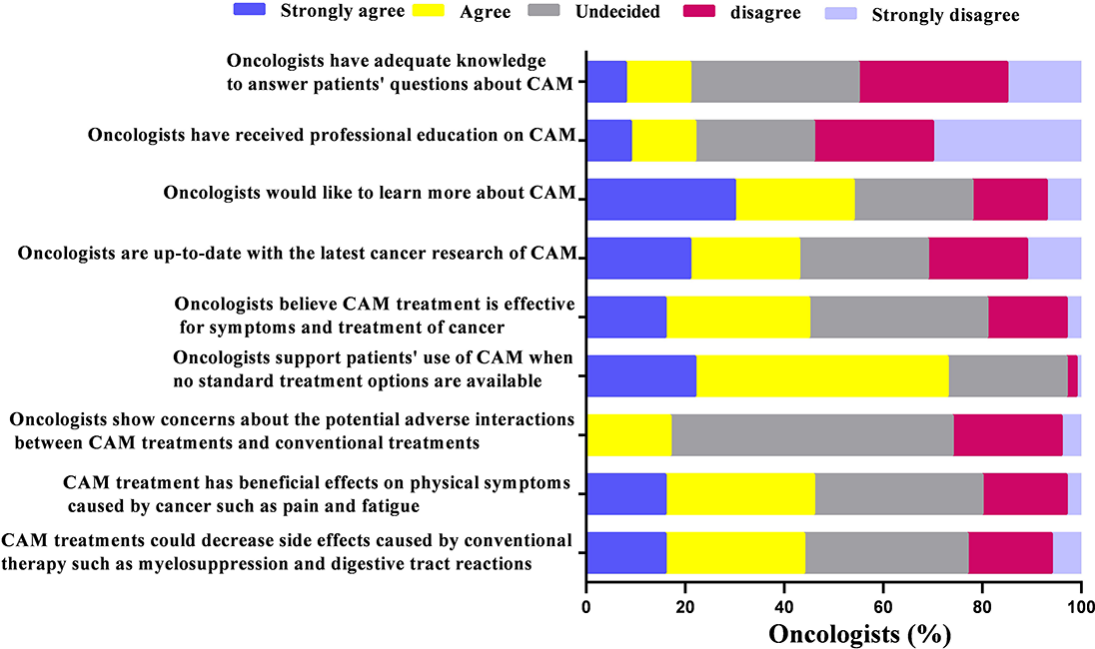
Oncologists’ knowledge and opinion patterns about complementary and alternative medicine (*N* = 6,007)

Four out of five of the oncologists self-reported inadequate knowledge to answer patients’ questions about CAM, meanwhile, only 22.0% of the 6007 oncologists reported receiving professional education on CAM. More than half (54.1%) expressed their willingness to learn more about CAM while 42.0% of them believed they would be up-to-date with the latest cancer research of CAM. When asked about the topics of CAM treatments, 44.9% of the oncologists believed CAM treatment was effective in cancer treatment and symptom control, and 73.1% of them supported patients’ use of CAM when no standard treatment options were available. A minority of oncologists (16.6%) showed concerns about the potential adverse interactions between CAM treatments and conventional treatments. When asked about the clinical benefits of CAM, about half of the oncologists (45.1%) agreed with the statement that CAM treatment has beneficial effects on physical symptoms caused by cancer such as pain and fatigue, as well side effects in patients treated with conventional therapy such as myelosuppression and digestive tract reactions (43.9%) (Figure 2).

Communication and practice patterns regarding CAM were analyzed using multivariate logistic regression model (Table 4). Oncologists’ mean age (≥ 33 years), gender, medical license for TCM, self-reported adequate knowledge and professional CAM education were found to be significantly associated with oncologists initiating discussion about CAM use, encourage CAM use for treatment, supporting patients’ use of CAM when no standard treatment options are available and believing CAM treatment was effective. Female oncologists were more likely to initiate discussion about CAM use. Oncologists who were in urban metropolises or academic hospital were more likely to encourage CAM use for treatment. Also, oncologists who were in academic hospital or had working years of over six years were more likely to supporting patients’ use of CAM and believe CAM treatment is effective. Results of the univariable analysis were summarized in Supplementary Table 1.

**Table 4:** Multivariable analysis of communication and practice patterns regarding CAM (*n* = 6,007)

Variable	Initiate discussion about CAM use		Encourage CAM use for treatment		Support patients’ use of CAM when no standard treatment options		Believe CAM treatment is effective for symptoms and treatment of cancer	
	OR	95% CI	OR	95% CI	OR	95% CI	OR	95% CI
Age (≥ 33 vs < 33 years)	**1.45**	1.25–1.69	**1.62**	1.38–1.91	**1.69**	1.44–1.98	**1.25**	1.04–1.40
Sex (female vs male)	**1.17**	1.03–1.34	1.08	0.94–1.25	1.03	0.90–1.19	1.03	0.90–1.18
Region								
Provincial capitals vs urban metropolises			**0.74**	0.61–0.90	0.96	0.80–1.15	**0.81**	0.68–0.97
Other cities vs urban metropolises			**0.80**	0.70–0.92	0.99	0.86–1.13	0.95	0.83–1.08
Practice setting								
General hospital vs academic hospital	0.86	0.68–1.10	**0.41**	0.32–0.52	**0.70**	0.54–0.92	**0.59**	0.47–0.75
Other hospital vs academic hospital	0.92	0.72–1.16	**0.59**	0.47–0.74	0.86	0.67–1.12	**0.69**	0.55–0.86
Working duration (≥ 6 vs < 6 years)	0.96	0.82–1.12	1.06	0.90–1.25	**1.25**	1.06–1.47	1.17	1.00–1.37
Type of medical license (traditional Chinese medicine vs clinical medicine)	**2.42**	1.90–3.10	**5.00**	4.09–6.12	**2.60**	1.95–3.35	**2.17**	1.77–2.66
Have adequate knowledge to answer questions about CAM (no vs yes)*	**0.48**	0.39–0.60	**0.38**	0.31–0.45	**0.42**	0.33–0.54	**0.30**	0.25-0.36
Receive professional education (no vs yes)*	**0.37**	0.30–0.44	**0.32**	0.26–0.38	**0.52**	0.43–0.64	**0.25**	0.21-0.29

CAM: complementary and alternative medicine; OR: odds ratio.

*From Likert-scale type of statements: response of strongly agree or agree meant yes, response of undecided, disagree or strongly disagree meant no.

## DISCUSSION

This is the first study to demonstrate that China's oncologists’ knowledge, attitudes and clinical practice regarding CAM use by a national online survey. In this study, we found that more than half of China's oncologists did not agree that CAM is useful and effective but they preferred to CAM use when no standard treatment options were available. This finding is consistent with studies from other Asian countries [7, 9].

It has been reported that approximately 68.7%–75% of cancer patients used CAM in the United States and 44.6% in Japan [17–19]. However, no national survey has been conducted in China before this study. The main reasons included difficulty in developing a cross-sectional study in such a big country with large population, and cancer treatment is not limited in cancer hospital or oncology clinics, with a population of 1.36 billion and annual number of 0.81 billion CAM visits [20]. Such a national survey could also be conducted by targeting cancer patients, which will be mainly focused on their attitudes and usage of CAM. But, it is difficult to launch such a national survey focused on cancer patients because of the large number of cancer patients (4.3 million new cancer cases in 2015) and associated high costs.

This study covered the oncologists who came from all of the 31 provinces in China, including Hongkong and Macau. One out of six (15.9%) of the oncologists involved held TCM medical licenses, similarly, 14.7% of all the physicians in China held TCM medical licenses according to 2014 China Health Statistics Yearbook [20]. In China, the average age of the physicians was 37 years old and practicing for 13.1 years [20]. This national survey of China's oncologists involved a group of relatively low-age oncologists with short working years, which were lower than those found by other similar studies, reporting an age of 40 to 48 years old and a working year of 9 to 18 years [7, 9, 10, 21]. This may be due to the fact that the younger oncologists access to the Internet and application on mobile phones more often than some of the elder oncologists who might not be either interested in or being able to search the Internet or application on the phone. As a national survey, it was planned to recruit the oncologists of different levels with a reasonable distribution. Given the survey was conducted by DXY, younger physicians were easier to be contacted with this website. Therefore, all the outcome data may not really reflect the attitudes and practice patterns of most practicing oncologists. However, most oncologists will retire at the age of 50–60 in China, and it was valuable for us to focus on these younger oncologists because they would be main force for cancer treatment for several decades.

Our study showed that oncologists discussed with only 36.5% of their patients about CAM and 38% of oncologists encouraged their patients to further discussion when asked about the topic. The proportion of discussion was lower than that found by earlier studies [10, 22]. In China, the current very short clinical encounter time and oncologists’ inadequate knowledge on CAM may lead to the lower proportion of discussions. Barriers to patient-physician communication about CAM are complicated and not limited to indifference of physicians, opposition to CAM use and less knowledge of CAM [9, 23–24]. In our study, only one fifth of the oncologists revealed that they did have enough knowledge on CAM or received professional education. Moreover, there are so many cancer patients in China and limited number of physicians available to patients with cancer. As a result, physicians spend little time in consulting with their patients, and there was not enough time for physicians to discuss with patient on the CAM use. Prior studies found that discussing CAM with patients may strengthen physician-patient relationship [5, 10]. Our study also indicated that oncologists who have adequate knowledge as well as professional education of CAM were more likely to initiate discussion about CAM use. Therefore, we suggest that oncologists be encouraged to learn various CAM therapies, to inquire about CAM use of patients and to raise awareness of potential benefits and risks associated with CAM therapies.

Generally speaking, herbal medicine is the most common CAM therapy for cancer patients in China, which is consistent with the findings in this study, while Americans prefer vitamins/minerals to herbs because of cultural differences [25, 26]. Chinese people have used herbal treatments for thousands of years.

Several surveys revealed that most oncologists experienced concurrent use of anticancer drugs with CAM products and they also believe that some CAM therapies are potentially harmful to their patients, particularly in patients undergoing chemotherapy, targeted or hormonal therapies [9–10]. However, in the current study, only 17% of the oncologists showed concerns about the potential adverse interactions, for it is generally agreed that most CAM products are safe in China. Besides, few studies have explored the prevalence of toxicities directly related to CAM products such as green tea, Chinese herb and formatted Chinese medicine during conventional treatments. However, no recent clinical studies have focused direct evidence for adverse interaction, including adverse outcomes, increased toxicities, or reduced the effectiveness of primary therapies [14, 27]. To resolve this discrepancy, more clinical research is needed to examine the potential benefit or adverse effect of the concomitant use of CAM products with anticancer drugs to help physicians to better discuss with patients the potential risks and benefits of the combination.

This study has some limitations. We face huge challenges to make a clear definition of oncologists due to the fact that a large number of hospitals in China do not clearly specify the duties of their clinical departments for cancer treatment. Thus, the definition of oncologist in this study may not be appropriate. The results of the survey may not represent views of all China's oncologists, as we only recruited 6,007 oncologists who completed the online survey by DXY, and the average age of oncologists in the survey is relatively young likely because we chose to conduct an online survey. Additionally, this survey represents a convenience sample. Besides, the perceptions and attitudes of China's cancer patients on CAM still remain unclear. Knowing the actual prevalence of CAM use in cancer patients might have strengthened the study, for oncologists’ estimates about the prevalence of CAM use were generally lower than estimates from studies of cancer patients.

Regardless of limitations, this study suggested that the minority of China's oncologists discuss with their patients about CAM, and most of the oncologists self-reported lack of knowledge on CAM. It also indicated that more than half of oncologists in China did not encourage CAM use. With an increasing prevalence of CAM use, great efforts should be made to provide China's oncologists and medical school students with latest knowledge on CAM and professional education to effectively and safely use CAM for their patients and to strengthen CAM education in undergraduate curriculum in medical schools and integrate CAM training into fellowship programs. In the near future, a study should be conducted to find the prevalence of CAM use in cancer patients in China, which would allow comparison of these studies.

## MATERIALS AND METHODS

### Data sources

A national online cross-sectional survey was conducted between May 2015 to August 2015 on the platform provided by DXY (www.dxy.cn), the largest medical and paramedical related website in China with over five million registered members. This includes over 1.3 million physicians, medical researchers, pharmacists, medical students, and nurses. Participants logged into the DXY website, entered the DXY survey system in the home page and then completed the online survey. New users to DXY needed to complete a new registration that DXY would check and verify the information of each applicant by email and telephone follow-up. If the information was not correct, the application would be rejected. Also, an email and a WeChat message were sent to all DXY relevant physicians to invite them to complete the survey. In addition, physicians could participate in the survey on a DXY APP or DXY WeChat in their mobile phones. To encourage physicians to the participation, both a token bonus and a DXY lottery were awarded to the physicians who completed this survey. This token bonus allowed them to download articles or shop online at the DXY website. The lottery prizes included an Apple Watch, stethoscope and other items. The study was approved by the institutional review board of the Second Military Medical University.

### Surveys

The survey was developed through a systematic review of the literature and by discussions with experienced surgical and medical oncologists. Prior to the commencement of the survey, the questionnaire was distributed to a group of experienced oncologists for review. Based on their feedback, the survey was further revised, questions reworded and response elements for clarity.

In the beginning, participants were required to provide demographic information, including age, specialty and years of practice. In this part, participants were asked two more questions: 1) what was your profession? (Physicians, pharmacists, medical students, nurses and others) 2) whether you have ever administered treatment to a cancer patient in the past three months? (Yes or no) The participants were excluded if either they were not physicians or they answered “No” to the second question. They were told to terminate the survey at this point of the questionnaire. The National Center for Complementary and Integrative Health (NCCIH) defines CAM simply as health care approaches developed outside of mainstream Western, or conventional, medicine. Clearly the boundaries between CAM and conventional medicine (also called Western or allopathic medicine) are not absolute [28]. We defined Chinese herbal medicine, such as Chinese herbs and Chinese patent medicine in the survey as following: “the majority of treatments in TCM, including personalized decoctions with single herbs or mixtures and extracted condensed pills or capsules with Chinese herbs”. The survey included 29 questions, which was composed of the following four sections: 1) The characteristics of survey respondents. 2) Oncologists’ communication patterns with patients 3) Oncologists’ clinical practice patterns with patients. 4) Oncologists’ knowledge and opinion patterns about CAM. In this part, participants indicated their agreements (Likert-scale type: strongly agree, agree, undecided, disagree, or strongly disagree) with nine statements about CAM.

### Statistical analysis

Any incomplete questionnaire was excluded from the study by DXY, and all of the original data were provided by DXY. The data were then coded and checked for errors by two of the authors (G.L.Y and H.Q.Z). Descriptive statistics (frequency distribution, mean ± SD, and median) were used to summarize oncologists’ characteristics and outcome variables. The chi-square tests were performed to explore associations between oncologist characteristics and CAM practice patterns by univariate analysis, using all significant predictor variables. Multivariable logistic regression models were used to determine whether associations persisted after controlling for demographics and other relevant factors. Model building began with all variables having a *P value* < 0.25 from the chi-square tests. A *P value* cut-off of < 0.10 to enter and < 0.05 to remain in the model were used. Age and sex were kept in the model regardless of their significance. Once the list of variables to be used in our final model was selected, the functional form of each variable and multicollinearity between the variables were examined. All analyses were performed using SAS software (version 9.4, SAS Institute, Cary, NC, USA).

## SUPPLEMENTARY MATERIALS TABLE


