# Supplementary material for: National survey of China's oncologists' knowledge, attitudes, and clinical practice patterns on complementary and alternative medicine
Source: Oncotarget. 2017 Jan 9;8(8):13440–9. doi: 10.18632/oncotarget.14560 (PMC5355110; doi:10.18632/oncotarget.14560)
Supplement: Supplementary file 1 [file oncotarget-08-13440-s001.pdf]

National survey of China's oncologists' knowledge, attitudes, and clinical practice patterns on complementary and alternative medicine

Supplementary Materials

Supplementary Table 1: Univariable analysis of communication and practice patterns regarding CAM (*n* = 6,007)

Variable	Initiate discussion about CAM use		Encourage CAM use for treatment		Support patients' use of CAM when no standard treatment options		Believe CAM treatment is effective for symptoms and treatment of cancer	
	Yes (%)	*P**	Yes (%)	*P**	Yes (%)	*P**	Yes (%)	*P**
Age, years		< 0.001		< 0.001		< 0.001		< 0.001
< 33	2,019 (63.4)		963 (30.2)		2,126 (66.7)		1,303 (40.9)	
≥ 33	2,017 (71.5)		1,195 (42.4)		2,264 (80.3)		1,397 (49.5)	
Sex		0.087		0.790		0.548		0.445
Male	3,008 (66.6)		1,627 (36.0)		3,310 (73.3)		2,043 (45.2)	
Female	1,028 (69.0)		531 (35.6)		1,080 (72.5)		657 (44.0)	
Region		0.946		< 0.001		0.094		0.104
Urban metropolises	665 (67.4)		333 (33.7)		709 (71.8)		424 (43.0)	
Provincial capitals	1,567 (67.4)		765 (32.9)		1,675 (72.0)		1,026 (44.1)	
Other cities	1,804 (67.0)		1,060 (39.3)		2,006 (74.5)		1,250 (46.4)	
Practice setting		0.002		< 0.001		< 0.001		< 0.001
Academic hospital	1,572 (64.9)		689 (28.4)		1,659 (68.4)		982 (40.5)	
General hospital	2,150 (68.3)		1,237 (39.3)		2,384 (75.7)		1,469 (46.6)	
Other hospital	314 (72.4)		232 (53.5)		347 (80.0)		249 (57.4)	
Working duration, years		< 0.001		< 0.001		< 0.001		< 0.001
< 6	2,447 (65.4)		1,225 (32.7)		2,578 (68.9)		1,573 (42.0)	
≥ 6	1,589 (70.2)		933 (41.2)		1,812 (80.0)		1,127 (49.8)	
Type of medical license		< 0.001		< 0.001		< 0.001		< 0.001
Clinical medicine	3,179 (62.9)		1,384 (27.4)		3,512 (69.5)		1,936 (38.3)	
Traditional Chinese medicine	857 (89.8)		774 (81.1)		878 (92.0)		764 (80.1)	
Have adequate knowledge to answer questions about CAM[†]		< 0.001		< 0.001		< 0.001		< 0.001
Yes	1,070 (88.8)		900 (74.7)		1,104 (91.6)		996 (82.7)	
No	2,966 (61.8)		1,258 (26.2)		3,286 (68.4)		1,704 (35.5)	
Receive professional education[†]		< 0.001		< 0.001		< 0.001		< 0.001
Yes	1,168 (88.3)		930 (70.3)		1,173 (88.7)		1,071 (81.0)	
No	2,868 (61.2)		1,228 (26.2)		3,217 (68.7)		1,629 (34.8)	

CAM: complementary and alternative medicine.
[*]*P* was obtained from chi-square test.
[†]From Likert-scale type of statements: response of strongly agree or agree meant yes, response of undecided, disagree or strongly disagree meant no.
